# Assessment of the Anti-Inflammatory Effectiveness of Diclofenac Encapsulated in Chitosan-Coated Lipid Microvesicles in Rats

**DOI:** 10.3390/pharmaceutics17050607

**Published:** 2025-05-03

**Authors:** Ana-Maria Raluca Pauna, Liliana Mititelu Tartau, Alin Mihai Vasilescu, Angy Abu Koush, Ruxandra Teodora Stan, Marius Constatin Moraru, Cosmin Gabriel Popa, Liviu Ciprian Gavril, Roxana Florentina Gavril, Dragos Valentin Crauciuc, Ana Marina Radulescu, Cristinel Ionel Stan

**Affiliations:** 1 Department of Anatomy, Faculty of Medicine, “Grigore T. Popa” University of Medicine and Pharmacy, 700115 Iasi, Romania; paunaanamariaraluca@gmail.com (A.-M.R.P.); mariusmmc@gmail.com (M.C.M.); cosmingpopa@gmail.com (C.G.P.); l_gavril@yahoo.com (L.C.G.); roxi.sufaru@yahoo.com (R.F.G.); crauciuc.dragos@gmail.com (D.V.C.); anamarina_r@yahoo.com (A.M.R.); crististan00@gmail.com (C.I.S.); 2 Department of Pharmacology, Faculty of Medicine, “Grigore T. Popa” University of Medicine and Pharmacy, 700115 Iasi, Romania; maierean_angy@yahoo.com; 3 Department of General Surgery, “Grigore T. Popa” University of Medicine and Pharmacy, 700115 Iasi, Romania; alin.vasilescu@umfiasi.ro; 4 Department of Implantology and Prosthesis Technology, Faculty of Dental Medicine, “Grigore T. Popa” University of Medicine and Pharmacy, 700115 Iasi, Romania; stan_ruxi@yahoo.com

**Keywords:** diclofenac, lipid-based microvesicles, chitosan coating, anti-inflammatory effect, drug delivery

## Abstract

**Background**: Diclofenac (DCF) is a widely used nonsteroidal anti-inflammatory drug (NSAID), but its conventional formulations may have limited efficacy and are associated with adverse effects. This study aimed to evaluate the anti-inflammatory and antioxidant effects of diclofenac encapsulated in chitosan-coated lipid microvesicles (DCF-m) compared to free DCF in a rat model of subacute inflammation. **Method**: DCF-m was prepared using L-α-phosphatidylcholine and coated with chitosan (CHIT). Subacute inflammation was induced using the cotton pellet granuloma model, and animals were divided into four groups (*n* = 5): a negative control group without granuloma receiving vehicle (double-distilled water), a control group with granuloma receiving vehicle, a group with granuloma treated with 15 mg/kg of free DCF, and a group with granuloma treated with 15 mg/kg of DCF-m. **Results**: Both DCF and DCF-m significantly reduced granuloma mass, body weight gain, and serum inflammatory markers compared to the control group with granuloma. Moreover, DCF-m treatment led to a more pronounced reduction in granulomatous inflammation and a greater enhancement of antioxidant enzyme activity than free DCF. **Conclusions**: These findings suggest that DCF-m exhibits superior anti-inflammatory and antioxidant properties compared to conventional diclofenac in a model of subacute inflammation.

## 1. Introduction

Nanomedicine employs nanotechnology for the diagnosis, prevention, and treatment of diseases. Nanoformulations consist of carrier materials, usually in the form of submicrometer-sized nanoparticles, designed to improve the targeted delivery of encapsulated substances. This innovation enables more precise and efficient delivery of therapeutic agents to disease sites while reducing exposure to healthy tissues that may be affected [1]. The main goal of this technology is to achieve a better balance between treatment efficacy and potential toxicity [2].

Nanoparticles are colloidal systems with sizes ranging from 10 to 100 nm, primarily composed of biodegradable polymers or lipids that can effectively encapsulate active substances through sequestration or absorption within their polymer matrix. These nanoparticles can adopt a reservoir-like structure, typically featuring a liquid core surrounded by a thin polymeric membrane just a few nanometers thick. Lipid nanoparticles provide the additional advantage of avoiding polymer degradation. Depending on the specific polymers or lipids used, nanoparticles have shown significant utility in delivering active substances via parenteral, oral, or local administration, tailored to the needs of the therapeutic application [3].

These nanoparticles can be created from mixtures of liquid or solid lipids, supported by emulsifying agents. The selected lipids for nanoparticle construction are biocompatible and highly tolerated by the human body, including triglycerides, fatty acids, and steroids. The use of various emulsifying agents also enhances the stabilization of these nanosystems. Compared to other nanoformulations, lipid nanoparticles offer several advantages, such as cost-effectiveness, ease of large-scale production, biocompatibility, biodegradability of the materials, low toxicity potential, controlled and modified drug release capabilities, improved solubility of active substances, and the ability to solubilize both hydrophilic and lipophilic agents [4]. It is important to note that lipid nanoparticles are distinct from micro-emulsions, which maintain thermodynamic stability in dispersing lipids and water and rely on surfactants and co-surfactants for stabilization [5]. Key factors for characterizing lipid nanosystems include particle size and distribution within the colloidal solution, Zeta potential, polymorphism, degree of crystallization, extent of drug loading, and drug release profile.

Lipid vesicles are among the most widely utilized nanoparticles for drug transport and delivery. They not only enable sustained drug release but also reduce toxicity by minimizing exposure of sensitive tissues to harmful medications. Their versatility is enhanced by the ability to actively target specific sites through the attachment of site-specific ligands. Additionally, liposomes can deliver hydrophobic, hydrophilic, and amphipathic compounds, thereby improving their pharmacological efficacy [6,7].

Various lipid types have been utilized as carriers to improve drug solubility and gastrointestinal absorption, with cholesterol being a prominent component. Cholesterol is crucial for stabilizing lipid vesicles, increasing their rigidity, enhancing encapsulation efficiency, and slowing the release rate of active substances [8]. As carrier nanoparticles, liposomes exhibit important biological properties, including extended circulation time in the bloodstream, the ability to attach ligands to their surfaces for targeted delivery, contrast-enhancing capabilities, and sensitivity to various stimuli. These characteristics make liposomes particularly effective systems for pharmaceutical delivery [9].

Developing nanoparticulate systems for targeted drug delivery can be challenging, especially when attempting to retain agents with therapeutic potential, particularly for localized treatment of inflammation and pain.

Currently, non-opioid analgesics, such as antipyretic analgesics and non-steroidal anti-inflammatory drugs (NSAIDs), are among the most frequently prescribed medications globally. NSAIDs are a class of pharmaceutical compounds that provide analgesic and anti-inflammatory effects by inhibiting cyclooxygenases (COX). Prominent NSAIDs, including naproxen, ibuprofen, and DCF sodium, are commonly used to treat conditions such as rheumatoid arthritis and ankylosing spondylitis, often requiring prolonged administration. However, COX-1 inhibition may lead to severe gastrointestinal irritation, while COX-2 inhibition is associated with cardiovascular complications. Consequently, long-term use of these medications can result in adverse effects, including peptic ulcers, gastrointestinal bleeding, and tachycardia [10].

DCF is a monocarboxylic acid derived from phenylacetic acid and features a (2,6-dichlorophenyl)amino group at position 2. It can be classified as a secondary amino compound, an amino acid, a dichlorobenzene, an aromatic amine, and a monocarboxylic acid. Its origin is linked to phenylacetic acid and diphenylamine, and it exists as the conjugate acid of DCF(1-). DCF falls into Class II of the biopharmaceutical classification system, characterized by high permeability but low solubility [3]. As a derivative of phenylacetic acid, DCF shares structural and pharmacological similarities with mefenamic acid and sodium meclofenamate [3,11].

The action mechanism of DCF involves the inhibition of lipoxygenases and the activation of the antinociceptive nitric oxide–guanosine monophosphate pathway. There are also theories proposing that it may inhibit phospholipase A2. These additional effects likely contribute to DCF’s remarkable potency, making it one of the most effective NSAIDs available [12]. In terms of pain relief, DCF shows exceptional strength, being six times more potent than indomethacin, sulindac, and codeine, fifteen times more effective than naproxen, and more than forty times stronger than acetylsalicylic acid. However, its oral bioavailability is limited due to its low solubility in water and the acidic environment of the stomach. Approximately 50–52% of DCF is metabolized in the liver and undergoes enterohepatic circulation, with 35% concentrated in bile and the remaining 65% excreted in urine. Despite having a relatively long duration of action of 6–8 h, its half-life is relatively short, ranging from 1.5 to 2 h, necessitating administration in 3–4 daily doses [12].

The present study is situated at the intersection of nanomedicine and anti-inflammatory pharmacotherapy, aiming to address a critical clinical challenge: optimizing the efficacy and safety of widely used NSAIDs such as DCF. While the pharmacological efficacy of DCF is well established, the clinical limitations of its conventional use highlight the need for improved delivery systems. In this context, our study is positioned within a broader effort to refine anti-inflammatory therapies through nanotechnology-based approaches. Despite substantial progress in developing and characterizing nanoformulations, the literature reveals a critical gap in translational research, specifically, the in vivo validation of these systems in models that mimic the complexity of inflammatory responses. To address this, we employed a standardized subacute inflammation model to investigate both local and systemic effects of a novel chitosan-coated lipid microvesicle formulation of DCF. This approach allows us to evaluate not only anti-inflammatory efficacy, but also antioxidant capacity, offering a more holistic understanding of the therapeutic potential. By focusing on a clinically relevant delivery challenge and applying a robust experimental model, this study contributes meaningful insights to the field of drug delivery and inflammation management, without extending into results or interpretations prematurely.

While numerous studies have explored the development and physicochemical characterization of lipid- and polymer-based nanocarriers for NSAID delivery, most focus predominantly on in vitro release kinetics and formulation stability. Despite the promising potential of these systems, in vivo pharmacodynamic data remain scarce, particularly regarding the anti-inflammatory and antioxidant performance of DCF-loaded lipid vesicles in subacute inflammation models. Moreover, although chitosan-coated lipid systems have demonstrated enhanced encapsulation efficiency and prolonged drug release, their comparative biological effects against conventional DCF formulations are not well established. This deficiency in translational research limits our understanding of the real-world therapeutic benefits such systems may offer.

Al-Lawati et al. provided a comprehensive review of preclinical efforts in the development of nanodelivery systems for NSAIDs, with particular focus on their pharmacokinetic and pharmacodynamic properties. The authors emphasized that, despite encouraging in vitro results, there remains a significant shortage of in vivo studies assessing the anti-inflammatory and antioxidant efficacy of these nanoformulations in validated subacute inflammation models, such as the cotton pellet granuloma test [13].

Similarly, other researchers investigating nanoparticle-based drug delivery systems for the treatment of knee osteoarthritis noted that, although numerous studies have explored such technologies, only a limited number have thoroughly evaluated their in vivo anti-inflammatory and antioxidant effects. This review highlighted a pressing need for more comprehensive in vivo assessments to bridge the existing gap between preclinical innovations and clinical applications [14].

Specifically regarding DCF, Boarescu et al. examined the combined effects of curcumin nanoparticles and DCF sodium on local edema and oxidative stress parameters in a rat model of acute inflammation. Their findings demonstrated a significant improvement in antioxidant markers when curcumin nanoparticles were co-administered with DCF. However, the study also underscored the necessity for additional in vivo research to validate these results and to investigate the long-term safety and therapeutic efficacy of such nanoformulation strategies [15].

Although considerable progress has been made in developing and characterizing nanoformulations, the translation of these innovations into in vivo applications remains insufficient, particularly in models that accurately capture the complexity of inflammatory responses.

Our study addresses this critical shortfall by evaluating both local and systemic anti-inflammatory responses and oxidative stress modulation in vivo, using a standardized granuloma model to assess the functional impact of nano-encapsulated DCF.

The aim of this study was to investigate the local and systemic anti-inflammatory and antioxidant potential of DCF sodium formulated in chitosan-coated lipid microvesicles (DCF-m), using the cotton-pellet-induced granuloma model in rats as a representative model for subacute inflammation.

We hypothesized that the DCF-m formulation would provide superior anti-inflammatory and antioxidant effects due to enhanced bioavailability, sustained drug release, and improved targeting of inflammatory tissues. This hypothesis reflects our expectation that nanoencapsulation would mitigate the limitations of conventional DCF administration and offer a more effective and safer alternative for inflammatory disease management.

Our objectives were: (1) to evaluate the local anti-inflammatory effects of DCF-m by quantifying granuloma formation, a hallmark of subacute tissue inflammation characterized by cellular infiltration and fibrous tissue proliferation; (2) to assess the systemic impact of the treatment by analyzing serum inflammatory markers and granulomatous tissue structure, offering insights into the formulation’s capacity to modulate inflammation beyond the site of induction; (3) to investigate the influence of DCF-m on oxidative stress-related parameters, including the activity of endogenous antioxidant enzymes, as oxidative imbalance is a key component of the inflammatory cascade.

## 2. Materials and Methods

### 2.1. Preparation and Characterization of DCF-m

#### 2.1.1. Substances

DCF sodium (CAS number—287840), along with the materials necessary for microparticle preparation, specifically L-alpha-phosphatidylcholine (CAS number P5638), cholesterol (CAS number—C8667), chitosan (CHIT) (CAS number—C3646), and chloroform (CAS number—C2432), were obtained from Sigma-Aldrich Chemical Co. (Steinheim, Germany) (https://www.sigma-aldrich.com—accessed on 12 of March 2025). The lipid employed was L-alpha-phosphatidylcholine, type XVI-E, which was approximately 99% pure. The CHIT used in this study had a degree of N-deacetylation of 79.7%, an average molecular weight of Mw = 310,000 g/mol, and a polydispersity index of 3.26. A 0.5% (*w*/*w*) CHIT solution was prepared using a 0.5% (*v*/*v*) acetic acid solution. Distilled water was sourced from Sicomed in Romania.

#### 2.1.2. Obtaining of DCF-m

**Figure 1:**
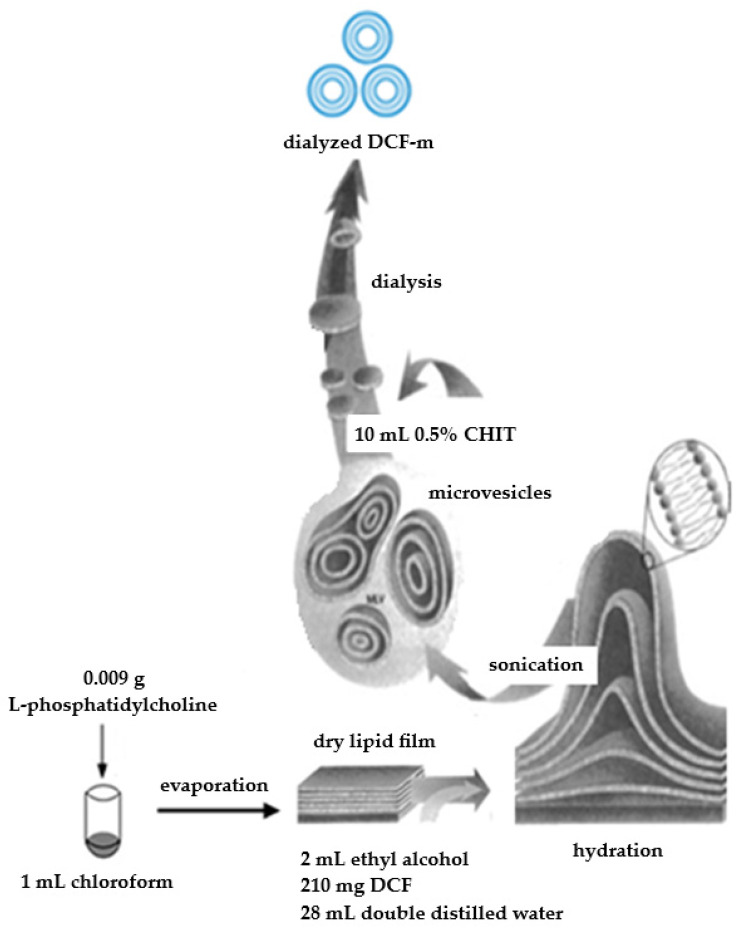
Schematic illustration of the DCF-m preparation.

The DCF-m were formulated using the thin-film hydration method, followed by CHITn deposition to enhance stability. Initially, 0.009 g of lipid L-alpha-phosphatidylcholine were dissolved in 1 mL of chloroform, which was then evaporated using a Rotary Evaporator RE-2000A (Ya Rong Biochemical Instrument Factory, Shanghai, China). The resulting lipid film was vacuum-dried for two hours (Figure 1).

Subsequently, a solution of 210 milligrams of DCF in 2 mL of ethyl alcohol, and 28 mL of double distilled water was prepared and stirred magnetically for two hours. This solution was then used to hydrate the dry lipid film, followed by ultrasonic treatment at 25% amplitude for 25 min, delivering 20,000 kJ at 25 °C with a Bandelin 2450 SONOPULS ultrasonic homogenizer (Sigma-Aldrich Chemical Co., Steinheim, Germany) (Figure 1).

For vesicle coating, 10 mL of a 0.5% CHIT solution was added to the drug-loaded microparticle suspension and magnetically stirred at 800 rpm for ten minutes (Figure 1). To approximate physiological pH, the vesicle suspensions underwent dialysis for two hours using double distilled water. Dialysis was conducted with tubular fiber membranes (D6191-25EA, 12,000 Da MWCO, Sigma-Aldrich Chemical Co., Steinheim, Germany).

The obtained DCF-m were previously characterized physicochemically, with encapsulation efficiency assessed. Additionally, in vitro release and hemocompatibility studies were conducted, while in vivo biocompatibility was evaluated [16].

### 2.2. In Vivo Experimental Researches

#### 2.2.1. Ethics of the Research

The experimental protocol received approval from the “Research Ethics Committee of UMF “Grigore T. Popa” from Iaşi” (Ethical Approval No. 24/21.12.2020) and was also authorized by the Veterinary Health Directorate (DSV authorization for project No. 32/1.03.2021). The research methodology was designed in complete accordance with national regulations [17] as well as European legislation [18] governing experiments on laboratory animals.

#### 2.2.2. Animals

The study was conducted at the CEMEX laboratories of the “Grigore T. Popa” University of Medicine and Pharmacy, using animals sourced from the “Cantacuzino” National Military Medical Institute for Research and Development, Baneasa Station, Bucharest, Romania.

Twenty healthy, genetically unaltered, pathogen-free white Wistar rats (8 weeks old, 200–250 g) were randomly selected for the experiment. The animals were housed individually under controlled conditions: a temperature of 21 °C ± 2 °C, relative humidity of 60–70%, and a 12 h light–dark cycle. They had unrestricted access to water and standard food, except during the experimental period.

To minimize chronobiological interference, tests were conducted during the light phase, between 8 a.m. and 12 p.m. daily. At the conclusion of the study, the rats were euthanized under general anesthesia with 3% isoflurane, following standard laboratory animal euthanasia protocols [19].

#### 2.2.3. Inflammatory Granuloma Test

**Figure 2:**
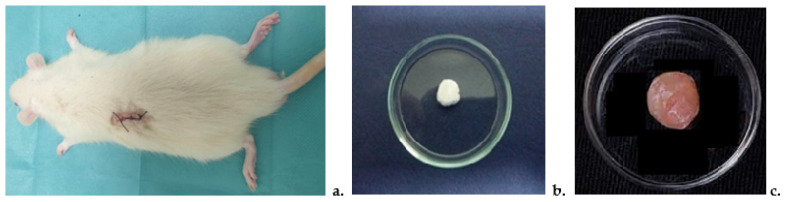
Granuloma test. (**a**) Rat with subcutaneous pellet implant; (**b**) sterile cotton pellet for subcutaneous implantation in rat; (**c**) inflammatory granuloma produced by subcutaneous cotton pellet implant.

The cotton pellet-induced granuloma model is the most effective method for evaluating drug efficacy across the transudative, exudative, and proliferative phases of subacute inflammation [20]. To assess the effects of DCF incorporated into microparticles (DCF-m), rats were divided into four groups (*n* = 5) and received intragastric administration via an eso-gastric tube: Group 1 (Negative Control): 0.3 mL of distilled water per 100 g body weight; Group 2 (Control): rats with granuloma receiving 0.3 mL of distilled water per 100 g body weight; Group 3 (DCF): rats with granuloma receiving DCF at 15 mg/kg body weight; Group 4 (DCF-m): rats with granuloma receiving DCF-loaded microparticles at 15 mg/kg body weight. On day one, animals were anesthetized with ketamine (80 mg/kg) and xylazine (10 mg/kg), followed by fur shaving and a posterior incision for subcutaneous implantation of sterile 62 mg cotton pellets (Figure 2a).

The implantation of cotton pellets (Figure 2b) induced inflammatory granulomas in all experimental groups, triggering a subacute inflammatory response that persisted for seven days. Acting as foreign bodies, the pellets led to fluid and protein accumulation, neutrophil, macrophage, and fibroblast infiltration, and small blood vessel proliferation, forming the characteristic reddish granulation tissue. This model is widely used to assess the transudative, exudative, and proliferative phases of subacute inflammation. The volume of fluid absorbed by the pellet directly impacts granuloma wet weight, while dry weight reflects the extent of granulomatous tissue formation. To minimize discomfort at the implantation site, benzocaine 20% spray was applied three times daily [21]. On day eight, animals were re-weighed and euthanized under 3% isoflurane anesthesia. The granuloma and surrounding granulation tissue (Figure 2c) were excised and dried at 60 °C until a constant weight was obtained. Granuloma mass was calculated by subtracting the post-experiment pellet weight from its initial baseline weight. Results were expressed as mean ± standard deviation (S.D.) for average animal weight and granuloma mass at each assessment point and for each tested substance.

#### 2.2.4. Blood Analysis

Blood samples were collected at 24 h and 7 days post-administration for hematological and biochemical analysis. At the study’s conclusion, animals were euthanized under isoflurane anesthesia, and tissue samples (liver, kidney, stomach) were harvested for histopathological evaluation.

For laboratory assessments, rats were anesthetized with 1% isoflurane, and a rapid, non-traumatic venous blood collection (0.3 mL) was performed from the lateral tail vein, enabling multiple samplings without causing distress [22,23,24]. Analyses included hematological parameters: white blood cell count (polymorphonuclear leukocytes, lymphocytes, eosinophils, monocytes, basophils), C-reactive protein (CRP), fibrinogen levels, interleukin-6 (IL-6), tumor necrosis factor-α (TNF-α), serum complement levels, and the phagocytic activity of peripheral blood PMNs.

Oxidative stress markers were assessed spectrophotometrically, measuring superoxide dismutase (SOD) activity via the xanthine/xanthine oxidase method [25], glutathione peroxidase (GPx) levels using the dithio-nitrobenzoic acid (DTNB) reagent [26], and malondialdehyde (MDA) concentration through the thiobarbituric acid method [27].

#### 2.2.5. Histopathological Evaluation

Following euthanasia, granulomas were harvested and immersed in a 10% formalin solution for 48 h to preserve cellular structure and prevent autolysis. After fixation, the samples were rinsed with phosphate-buffered saline to remove excess fixative and then dehydrated in a graded series of ethanol, starting with 70% ethanol and gradually increasing to 100% ethanol, with each step lasting approximately 30 min to ensure complete dehydration.

Once dehydrated, the samples were cleared in xylene, replacing the ethanol, to prepare the tissue for embedding. After clearing, the samples were infiltrated with paraffin wax at approximately 60 °C to facilitate embedding. The tissue was then oriented in a mold and allowed to cool, solidifying the paraffin around it. After solidification, the paraffin blocks were sectioned into thin slices (5 μm thick) using a semi-automatic precision microtome (CUT 5062 Microtome, Nieder-Olm, Germany). The sections were mounted onto glass slides, deparaffinized in xylene, and rehydrated through a series of decreasing ethanol concentrations.

Finally, the tissue sections were stained with Masson’s trichrome stain to highlight cellular components and structures within the granulation tissue. The slides were then examined using a Nikon Ti-E Eclipse optical microscope (Nikon, Tokyo, Japan), equipped with a Nikon Coolpix 950 camera (optical zoom ×3, 1600 × 1200 resolution, 1.92 Mpx).

#### 2.2.6. Data Analysis

The results are presented as mean values with standard deviations (S.D.) for each measurement time point. Data were analyzed using SPSS version 24.0 for Windows, ANOVA one-way method, and Statistics Toolbox in Matlab version 9.8.0.132.3502—2020a, (The MathWorks, Inc., Natick, MA, USA), with appropriate statistical functions employed to assess data distribution. To facilitate detailed comparisons, post hoc analyses using the Newman–Keuls and Tukey tests were conducted. Statistical significance was defined as *p*-values less than 0.05 when compared to the control group.

## 3. Results

Our previous study reported the development of novel lipid-based microvesicles encapsulating DCF, with an average size of 562 ± 13.37 nm, with a polydispersity index of 0.189, and a Zeta potential of 45 ± 1.67 mV, indicating excellent colloidal stability (Table 1).

**Figure 3:**
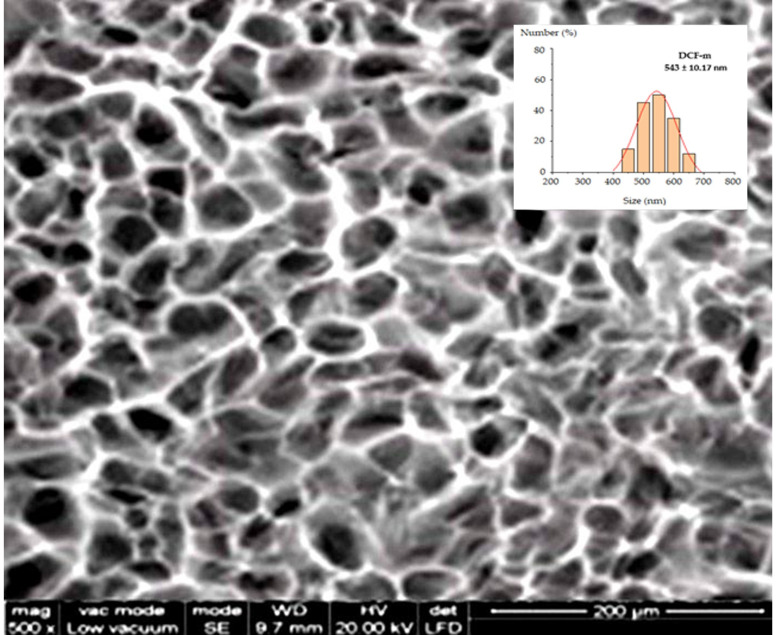
Scanning electron microscopy (SEM) micrograph of DCF-m at 2000×.

The SEM image (acquired using the EDAX-Quanta 200 system, Eindhoven, Germany) reveals that the individualization of the vesicles significantly enhances the delivery of active substances, with each vesicle acting as an independent entity, thereby promoting a more uniform distribution within the biological system. The DCF-m display a notably homogeneous and smooter surface, indicating enhanced stability and a heightened resistance to external interferences (Figure 3). Image analysis was performed using the widely recognized ImageJ software, version 1.53, revealing that the vesicles exhibited a size distribution with an average diameter of 543 ± 10.17 nm (Figure 3).

The encapsulation efficiency was 79.4% [13]. UV-Vis analysis revealed a peak absorption at 276 nm, with no interference from solvents or polymeric components. In vitro release studies demonstrated sustained drug release from the chitosan-stabilized vesicles, with 8.2% of DCF released at 30 min, 39.6% at 90 min, and a maximum release of 93.6% after 6 h [13]. Additionally, in vitro hemocompatibility and in vivo biocompatibility studies in rats showed no significant alterations in blood biochemical and immunological parameters, oxidative stress markers, or liver and kidney histology, further supporting the safety profile of these microvesicles [13].

### 3.1. Changes in Animal Weight and Granuloma Mass

Seven days after subcutaneous pellet implantation, a significant increase in animal weight (## *p* < 0.01) was observed compared to baseline, likely due to the development of subacute inflammation. Treatment with DCF resulted in a notable (** *p* < 0.01) reduction in body weight relative to the Control group. Animals treated with DCF-m exhibited a more pronounced (** *p* < 0.01) weight decrease after seven days compared to the Control group (Table 2). The weight-reducing effects of DCF-m were notably greater than those of DCF one week post-granuloma induction (Table 2). These findings suggest that while subcutaneous pellet implantation induces weight gain due to subacute inflammation, both DCF and DCF-m effectively counteract this effect, with DCF-m showing superior efficacy in weight reduction (Table 2).

Regarding granuloma mass, significant changes were observed. Seven days post-inflammation induction, granuloma mass increased substantially (## *p* < 0.01) compared to baseline (Table 2). DCF treatment led to a significant (** *p* < 0.01) reduction in granuloma mass compared to the Control group. Treatment with DCF-m resulted in a marked (** *p* < 0.01) decrease in granuloma weight relative to the Control group. Notably, DCF-m exhibited a more pronounced reduction in granuloma weight than DCF after seven days of treatment (Table 2). These results highlight that while subacute inflammation significantly increases granuloma mass, both DCF and DCF-m are effective in reducing it, with DCF-m displaying a more robust effect.

### 3.2. Blood Parameters

Following the application of subcutaneous pellets, a significant increase in PMN percentage was observed in the Control group at day 7 compared to baseline (## *p* < 0.01). One week after DCF administration, the PMN percentage showed a noticeable reduction (* *p* < 0.05) compared to the Control group. Treatment with DCF-m led to a significant decrease (* *p* < 0.05) in PMN percentage relative to the Control group after one week (Table 3).

Seven days after the induction of subacute inflammation, the Ly percentage in the pellet Control group decreased considerably compared to baseline (## *p* < 0.01). One week following DCF administration, the Ly percentage increased significantly (* *p* < 0.05) compared to the Control group. Similarly, DCF-m treatment resulted in a significant increase (* *p* < 0.05) in Ly percentage relative to the pellet Control group after 7 days (Table 3).

No notable variations were observed in the percentages of E, M, and B among the groups that received DCF, DCF-m, and the Control group one week post-pellet implantation (Table 3).

**Figure 4:**
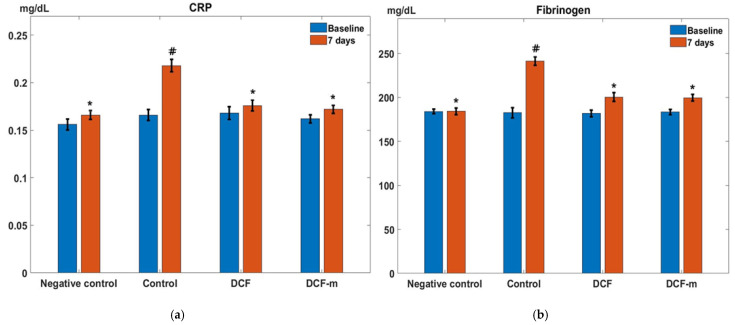
Influence of DCF and DCF-m on serum CRP (C-reactive protein) (**a**) and fibrinogen (**b**) levels in cotton pellet granuloma test in rats. * *p* < 0.05 versus Control, # *p* < 0.05 versus baseline. Data are expressed as mean ± S.D. of mean for five rats in a group.

In the Control group, subacute inflammation led to a significant increase in serum CRP levels at day 7 compared to baseline (# *p* < 0.05, confidence interval—95% CI for the difference: [0.03, 0.07]). One week after DCF administration, blood CRP levels significantly decreased (* *p* < 0.05, 95% CI for the difference: [−0.06, −0.02]) compared to the Control group in this experimental model of subacute inflammation in rats. Treatment with DCF-m resulted in a substantial reduction (* *p* < 0.05, 95% CI for the difference: [−0.06, −0.03]) in serum CRP levels relative to the Control group at day 7. The CRP-lowering effect of DCF-m was more pronounced than that of DCF, although the difference between the two groups was not statistically significant. (Figure 4a).

In the granuloma Control group, a significant rise in serum fibrinogen levels was observed one week after the experiment began (# *p* < 0.05, 95% CI for the difference: [43.71, 74.09]). Administration of DCF led to a marked reduction (* *p* < 0.05, 95% CI for the difference: [−55.11, −27.09]) in blood fibrinogen levels compared to the Control group at day 7 post-pellet implantation. At the same evaluation point, serum fibrinogen levels in the DCF-m-treated group showed a substantial decrease (* *p* < 0.05, 95% CI for the difference: [−54.05, −29.54]) relative to the Control group. DCF-m produced a greater decrease in fibrinogen levels compared to DCF, yet the difference did not reach statistical significance (Figure 4b).

**Figure 5:**
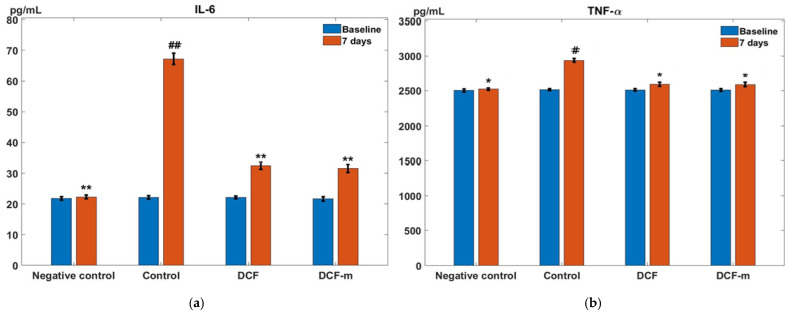
Influence of DCF and DCF-m on the serum level of IL-6 (interleukin 6) (**a**) and TNF-α (tumour necrosis factor alpha) (**b**) in inflammatory granuloma model in rats. * *p* < 0.05, ** *p* < 0.01 versus Control, # *p* < 0.05, ## *p* < 0.01 versus baseline. Data are expressed as mean ± S.D. of mean for five rats in a group.

In the Control group with induced granuloma, blood IL-6 levels showed a significant increase (## *p* < 0.01, 95% CI for the difference: [41.12, 49.04]) after 7 days compared to baseline. One week after administration, DCF significantly reduced (** *p* < 0.01, 95% CI for the difference: [−39.22, −30.34]) IL-6 levels compared to the granuloma Control group in this experimental model of subacute inflammation in rats. Treatment with DCF-m led to a statistically significant decrease (** *p* < 0.01, 95% CI for the difference: [−40.33, −30.34]) in serum IL-6 levels relative to the Control group after 7 days. DCF-m exhibited a more effective reduction of IL-6 levels compared to DCF; however, the difference between the two treatments was not statistically significant in the experiment (Figure 5a).

After 7 days, the Control group exhibited a substantial rise (# *p* < 0.05, 95% CI for the difference: [388.97, 454.59]) in TNF-α levels compared to baseline. One week following DCF administration, blood TNF-α levels were significantly reduced (* *p* < 0.05, 95% CI for the difference: [−385.62, −302.70]) compared to the granuloma Control group. Similarly, DCF-m treatment resulted in a statistically significant decrease (* *p* < 0.05, 95% CI for the difference: [−393.29, −305.27]) in TNF-α levels relative to the Control group after one week. In this experimental model of subacute inflammation in rats, DCF-m had a more pronounced effect on reducing serum TNF-α levels than DCF; despite this, the difference was not statistically significant (Figure 5b).

**Figure 6:**
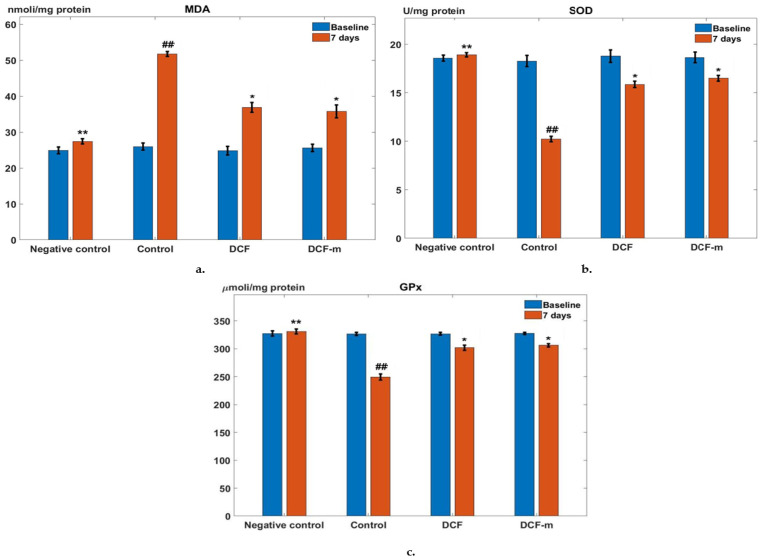
Influence of DCF and DCF-m on blood MDA (malondialdehyde) (**a**), SOD (superoxide dismutase) (**b**), and GPx (glutathione peroxidase) (**c**) levels in rats with granuloma. * *p* < 0.05, ** *p* < 0.01 versus Control, ## *p* < 0.01 versus baseline. Data are expressed as mean ± S.D. of mean for five rats in a group.

One week after granuloma formation, the Control group showed a significant increase in MDA activity (## *p* < 0.01, 95% CI for the difference: [23.37, 28.28]) compared to baseline. DCF administration led to a noticeable reduction (* *p* < 0.05, 95% CI for the difference: [−17.99, −11.77]) in serum MDA levels relative to the Control group at day 7. At the same evaluation point, MDA activity in the DCF-m group significantly decreased (* *p* < 0.05, 95% CI for the difference: [−18.85, −12.19]) compared to the granuloma Control group. The MDA-lowering effect of DCF-m was more pronounced than that of DCF in this experiment; nevertheless, the difference was not statistically significant (Figure 6a).

In the Control group, subacute inflammation resulted in a substantial decrease in serum SOD levels (## *p* < 0.01, 95% CI for the difference: [−9.37, −6.71]) after 7 days. Oral administration of DCF significantly increased (* *p* < 0.05, 95% CI for the difference: [4.74, 6.50]) SOD activity compared to the Control group at the same time point. Similarly, in the DCF-m group, a significant rise (* *p* < 0.05, 95% CI for the difference: [5.44, 7.08]) in blood SOD levels was observed compared to the granuloma Control group (Figure 6b).

At the 7-day evaluation, the granuloma Control group exhibited a marked reduction in blood GPx levels (## *p* < 0.01, 95% CI for the difference: [−89.31, −65.25]) compared to baseline. Treatment with DCF significantly enhanced (* *p* < 0.05, 95% CI for the difference: [38.06, 66.62]) GPx activity relative to the Control group after one week. Likewise, in the DCF-m group, serum GPx levels showed a notable increase (* *p* < 0.05, 95%CI for the difference: [44.53, 68.99]) compared to the granuloma Control group (Figure 6c). The effect of DCF-m in enhancing SOD and GPx activity was stronger than that induced by DCF after 7 days of the experiment, although the difference did not reach statistical significance (Figure 6b,c).

### 3.3. Histopathological Findings

**Figure 7:**
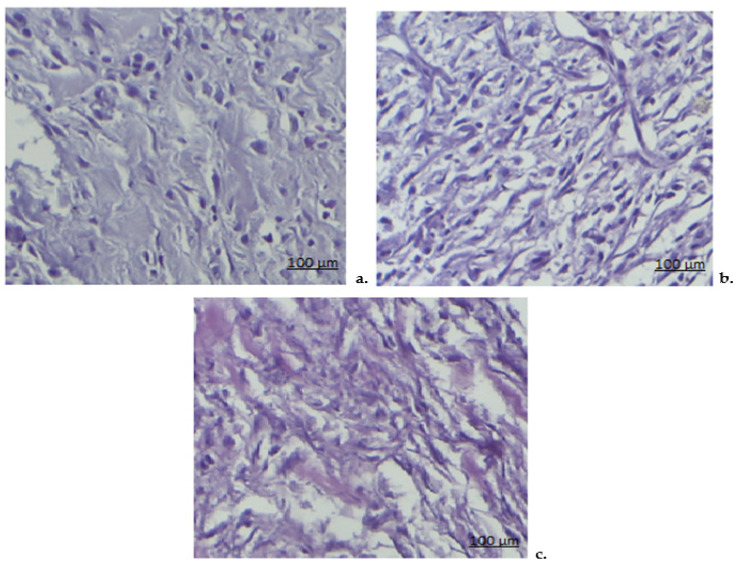
The structure of the granulation tissue in the Control group (**a**), DCF group (**b**), and DCF-m group (**c**). Masson trichrome stain ×20.

The subcutaneous implantation of sterile cotton pellets triggered the development of an inflammatory granuloma. In the initial stages, fluid- and protein-rich material accumulated, accompanied by the influx of macrophages, neutrophils, fibroblasts, increased collagen production, and the formation of small blood vessels. These processes together formed granulation tissue, a vascularized, reddish mass. Histopathological analysis of the granulomas in the Control group, where sterile cotton pellets were implanted, revealed a foreign body inflammatory reaction. This was characterized by a protein-rich exudate and a high presence of fibroblasts, neutrophils, and macrophages. The granuloma was surrounded by a well-vascularized fibrous capsule, with dispersed collagen fibers and numerous dilated blood vessels (Figure 7a).

In the DCF group, histological examination indicated a moderate reduction in the subcutaneous inflammatory response around the implanted pellet. This included a decrease in the infiltration of inflammatory cells, smaller blood vessels, and a reduced density of collagen fibers (Figure 7b).

In animals treated with DCF-m, there was a significant reduction in the local subacute inflammatory reaction, with a marked shrinkage of the granulomatous area. Fewer inflammatory cells and collagen fibers were seen, and the granuloma was encased by a collagen-rich connective tissue layer, while the exudative area within was notably smaller (Figure 7c).

## 4. Discussions

The development of nanocarriers for drugs, utilizing polymers, lipids, and biomaterials, has significantly advanced precision medicine by enabling the delivery of poorly soluble compounds and the customization of dosages [5,28]. However, research indicates that the nanoscale size of these carriers may induce novel host responses distinct from those triggered by larger systems made from the same biomaterials or by the unencapsulated drugs themselves. Consequently, a specific regulatory framework is necessary to establish scientifically robust and safety-focused guidelines, ensuring reliable biocompatibility assessments for these new nanodevices.

Lipid-based carriers, which are both biocompatible and biodegradable, offer a promising system for drug delivery. Nonetheless, they face limitations due to the potential for rapid elimination of the encapsulated drug following in vivo administration. To address this, modern technological processes have been employed to enhance stability by coating these vesicles with polymers, which prolongs their circulation in the bloodstream and supports sustained drug release [29,30].

A key area of research is the development of methods for incorporating NSAIDs, widely prescribed to manage pain, fever, and inflammation, into nanoparticle carriers. NSAIDs vary in pharmacokinetic, pharmacodynamic, and toxicological properties, which creates substantial interest in novel NSAID formulations through nanoparticle incorporation. While several studies have explored NSAID-loaded nanoparticles, there is limited information on their pharmacodynamic behavior in various inflammatory conditions [29]. Characterization of these carrier systems has demonstrated effective, sustained release in vitro [31,32,33], though data on their in vivo effects remain inconsistent [34].

Certain formulation variables and the choice of polymer(s) used in these NSAID delivery systems may offer pharmacodynamic advantages, particularly in mitigating the adverse effects of the active compound [29,35,36].

Research indicates that using drug carriers in the form of micrometer-sized vesicles can reduce the toxicity of active compounds [37,38,39]. Multiple studies have demonstrated that NSAID nanoparticles, in particular, offer the benefit of markedly reducing renal and gastrointestinal side effects commonly associated with the unencapsulated drug [31,40,41].

DCF sodium is a salt derived from a weak acid with a pKa value of 4. Its partition coefficient (n-octanol/aqueous buffer at pH 7.4) is 13.4, and it is chemically classified as 2-[(2,6-dichlorophenyl)amino]benzene acetic acid sodium salt [3] or o-(2,6-dichloroanilino)phenyl sodium acetate [42]. To reduce the frequency of doses for a drug with a short half-life, sustained-release formulations are needed. This approach aims to minimize the need for multiple doses, particularly because oral administration is often associated with gastrointestinal issues such as bleeding and ulcers [43].

Due to its amphiphilic nature, DCF tends to form clusters with cellulose, prompting the exploration of various formulation techniques to mitigate its side effects. Encapsulating drugs with well-defined structures is particularly advantageous in terms of absorption and targeted action within the body. One strategy to enhance these pharmacokinetic and pharmacodynamic properties is encapsulation in phospholipids. However, it has been suggested that DCF may induce structural changes in phospholipids, resulting in the formation of surface-active monomers [44].

Lipid-based formulations have demonstrated a significant ability to enhance the bioavailability of hydrophobic drugs like DCF when compared to traditional dosage forms [45]. The surface epithelium of the gastrointestinal tract is coated with mucus, which contains a phospholipid adsorbent layer, creating a hydrophobic zone between the epithelium and the luminal contents. Within this zone, various lipid species, similar to surface-active phospholipids, are present, with phosphatidylcholine being an ideal candidate for DCF incorporation. It has been observed that when this complex is surrounded by an ionic surfactant shield, it retains its lipophilic characteristics [46]. The development of extended-release formulations for DCF sodium aims to enhance the drug’s safety profile while offering the convenience of once-daily dosing for patients with chronic pain [11]. To minimize potential side effects associated with prolonged-release oral administration, considerable efforts are being made to develop nanoparticles capable of incorporating the drug over an extended period.

To improve DCF`s pharmacokinetics, pharmacodynamics, and minimize adverse reactions, especially gastrointestinal, various microparticle formulations have been developed using materials such as poly(lactic-co-glycolic acid), ethyl cellulose, chitosan/poly(methacrylic acid), poly-pyrrole/menthol, polyvinyl alcohol, and didodecyldimethylammonium bromide [47,48,49,50].

Extended-release formulations of DCF sodium salts have also been created to enhance their safety profile and enable convenient once-daily dosing, a useful approach for managing chronic pain [51]. In efforts to prevent the adverse effects associated with oral delivery of these sustained-release formulations, researchers have developed microparticles encapsulating DCF. Due to DCF’s amphiphilic nature, it tends to form cellulose aggregates, leading to innovative formulation techniques aimed at minimizing the drug’s known side effects [52].

Lipid-based formulations have proven effective in enhancing the bioavailability of hydrophobic drugs like DCF compared to traditional dosage forms [53]. Studies further show that DCF microspheres formulated with sodium alginate through ionotropic gelation or with lactic-co-glycolide have improved DCF’s release profile both in vitro and in vivo [54]. Another study highlighted increased absorption of DCF when incorporated into polyethyleneimine-functionalized sodium alginate/nanocrystalline cellulose/polyvinyl alcohol microspheres [55]. While these liposomes have been thoroughly characterized with respect to in vitro drug release, no data currently exist on their effects in laboratory animals.

Researchers have also developed sodium DCF thin lamellar vesicles using a thin film hydration method with soy lecithin, cholesterol, and solvent mixtures of ethanol, chloroform, or methanol. Although these nanosystems have been well-characterized in terms of physicochemical and structural properties, and drug release behavior, their effects in animal models remain unexplored [56]. Goh and colleagues created DCF liposomes using the pro-lipo-duo technique with dimethyl sulfoxide as a solvent. When administered orally, these DCF microparticles exhibited stronger anti-inflammatory effects than the unincorporated drug in carrageenan and formalin-induced paw inflammation tests, as well as in the subcutaneous pellet implant model in rats. This enhanced activity is thought to result from the liposomal delivery system’s ability to modify the drug’s bio-pharmacological properties, including increased solubility, improved lymphatic absorption, prolonged gastric transit, and additional protective effects, such as reduced metabolic degradation and slower gastric clearance [57].

While the previous study [16] did not address the pharmacodynamic effects of the formulation, the present work investigates anti-inflammatory efficacy in vivo, comparing DCF-m to conventional DCF. This enables us to assess the functional impact of the novel delivery system beyond biocompatibility.

Together with our previous work, this research contributes to a comprehensive evaluation of the DCF-m system, starting from formulation and safety profiling to preclinical therapeutic validation, which is essential for translational potential.

The present study compared the anti-inflammatory effects of lipid-based microparticles encapsulating DCF using the granuloma model. The cotton-pellet-induced granuloma model was selected because it is a standardized, well-established, and widely accepted model for evaluating the efficacy of anti-inflammatory agents in subacute inflammation. This model is commonly used in preclinical research to assess the proliferative phase of inflammation, which involves cellular infiltration, fibroblast proliferation, and collagen formation, all contributing to granuloma tissue development. It is particularly suitable for testing compounds intended to modulate chronic and subacute inflammatory responses in rodents, offering a reproducible and quantifiable method for comparing the anti-inflammatory potential of different formulations.

After one week of subcutaneous pellet implantation, experimental animals exhibited weight gain, consistent with subacute inflammation development. Treatment with DCF-m resulted in a statistically significant reduction in weight compared to controls, suggesting a therapeutic effect.

White blood count evaluation demonstrated that DCF-m effectively reduces neutrophil infiltration, supporting lymphocyte recovery without disrupting other immune cells (E, M, B). The significant CRP reduction suggests a lower inflammatory burden, while DCF-m exhibited greater efficacy than DCF in reducing fibrinogen levels, aligning with its other anti-inflammatory effects. Both DCF-m and DCF treatments significantly decreased IL-6 and TNF-α levels, with DCF-m showing superior efficacy. Since IL-6 and TNF-α are key pro-inflammatory mediators, their reduction highlights DCF-m’s role in cytokine modulation and immune homeostasis. This enhanced effect could be attributed to microvesicle encapsulation, which not only improves the bioavailability of the drug but also allows for more controlled and prolonged release at the site of inflammation. The prolonged release of DCF from DCF-m formulation may lead to sustained therapeutic levels over a longer period, reducing the acute inflammatory response more effectively. Although the differences observed between DCF-m and DCF did not reach statistical significance, the trend indicates that the microvesicle encapsulation may contribute to a more efficient and long-lasting anti-inflammatory action, potentially offering therapeutic advantages over conventional DCF.

DCF-m demonstrated a stronger ability to combat oxidative stress than DCF, reflected in reduced MDA levels and increased SOD activity, indicating lower lipid peroxidation and enhanced antioxidant defenses. This suggests a protective effect against oxidative damage, a key factor in chronic inflammation progression. The prolonged release of DCF within the microvesicles may facilitate a more continuous scavenging of reactive oxygen species (ROS), thus providing better protection against oxidative damage over time. This sustained antioxidant activity could be especially beneficial in chronic inflammatory conditions, where oxidative stress plays a key role in disease progression. Although the differences between DCF-m and DCF did not reach statistical significance, the data suggest that the extended release and improved delivery system of DCF-m may contribute to its stronger antioxidant effects.

Histological analysis confirmed that DCF-m treatment significantly reduced inflammatory cell infiltration and collagen deposition compared to DCF, highlighting its superior effect on granuloma tissue remodeling. By decreasing vascular dilation and granulation tissue formation, DCF-m demonstrated greater efficacy in resolving subacute inflammation.

## 5. Conclusions

This study explored the anti-inflammatory effects of a new approach using chitosan-coated lipid-based microvesicles to encapsulate DCF, and compared it to the use of free, unencapsulated DCF in a rat model of subacute inflammation. Think of DCF as the medicine and the microvesicles as tiny delivery vehicles, designed to transport the drug more efficiently to the site of inflammation. Both forms of DCF, free and encapsulated, were successful in alleviating inflammation, as shown by a decrease in key indicators of inflammation, such as inflammatory markers, exudate, and granuloma formation. Moreover, free DCF as well as DCF encapsulated in lipid microvesicles helped reduce oxidative stress that accompanies subacute inflammation in the rats. Using the cotton pellet granuloma model, the results revealed that DCF-m demonstrated a stronger combination of anti-inflammatory and antioxidant effects compared to the free DCF. This highlights how the microvesicle encapsulation can enhance the drug’s effectiveness, providing both better control over inflammation and protection against the oxidative damage caused by it. The development of these systems for encapsulating DCF, offers significant promise for enhancing the therapeutic efficacy of this widely used anti-inflammatory drug.

## Figures and Tables

**Table 1:** The average values size and Zeta potential of the investigated systems.

System	Size (nm)	Zeta Potential (mV)
DCF (solution)	3210 ± 21.45	−1.62 ± 0.05
uncoated DCF lipid vesicles	120 ± 8.15	−31.7 ± 1.03
DCF-m	562 ± 13.37	+45 ± 1.67

**Table 2:** Influence of DCF and DCF-m on the animal weight and granuloma mass ## *p* < 0.01 versus baseline, ** *p* < 0.01 versus Control. Data are expressed as mean ± S.D. of mean for five rats in a group.

Group	Mean Changes in the Animal Weight (g) After 7 Days	Dry Granuloma Mass (mg) at Baseline	Dry Granuloma Mass (mg) After 7 Days
Control	+7.3 ± 1.1 ##	62	112.8 ± 5.5 ##
DCF	+2.5 ± 0.7 **	62	69.3 ± 1.9 **
DCF-m	+2.2 ± 0.5 **	62	67.5 ± 3.7 **

**Table 3:** Influence of DCF and DCF-m on the white blood count (PMN—neutrophilic polymorphonuclear cells, L—lymphocytes, M—monocytes, E—eosinophils, B—basophils) components in rats with experimentally induced granuloma. * *p* < 0.05, ** *p* < 0.01 versus Control, ## *p* < 0.01 versus baseline. Data are expressed as mean ± S.D. of mean for five rats in a group.

		White Blood Count (%)				
		PMN	Ly	E	M	B
Negative control	baseline	17.67 ± 3.19	76.36 ± 8.41	2.37 ± 0.11	3.41 ± 0.09	0.19 ± 0.01
	7 days	17.83 ± 3.21 **	76.50 ± 7.93 **	2.19 ± 0.15	3.33 ± 0.13	0.15 ± 0.01
Control	baseline	17.45 ± 3.43	76.92 ± 8.55	2.21 ± 0.21	3.29 ± 0.15	0.13 ± 0.03
	7 days	35.33 ± 4.45 ##	58.79 ± 8.29 ##	2.42 ± 0.17	3.25 ± 0.21	0.21 ± 0.03
DCF	baseline	17.49 ± 3.67	76.84 ± 7.85	2.13 ± 0.13	3.37 ± 0.17	0.17 ± 0.01
	72 h	22.83 ± 4.51 *	71.32 ± 8.29 *	2.45 ± 0.21	3.21 ± 0.25	0.19 ± 0.05
DCF-m	baseline	17.31 ± 3.55	77.02 ± 8.19	2.33 ± 0.27	3.17 ± 0.33	0.17 ± 0.03
	7 days	22.55 ± 5.13 *	71.70 ± 8.67 *	2.15 ± 0.15	3.45 ± 0.27	0.15 ± 0.01

## Data Availability

The original contributions presented in this study are included in the article. Further inquiries can be directed to the corresponding author.
